# Functional Diseases of the Eye

**Published:** 1872-05

**Authors:** Swan. M. Burnett

**Affiliations:** Knoxville, Tenn., Sec. Knox Co. Med. Soc., and Med. Soc. E. Tenn.


### FUNCTIONAL DISEASES OF THE EYE.

BY SWAN. M. BURNETT, M. D., KNOXVILLE, TENN., SEC. KNOX CO. MED. SOC., AND MED. SOC. E. TENN.

Those affections of the eye that are commonly known as "Functional Diseases" are especially worthy of the attention of the practitioner, for two reasons:

1. Because of the certainty of relief by the adoption of proper means, and,

2. On account of the *completeness* of that relief in the majority of cases.

From the fact that some of these troubles are congenital, many patients suffering from them have but little idea of what perfect vision is, and when their defect is removed, a new world is, as it were, opened up to them, and beauties, hitherto undreamed of, are spread out before their astonished gaze. Their joy at the discovery is, therefore, very great, and, in some instances, has been known to manifest itself in shouts.

I call to mind the case of a prominent medical man in the State, who suffers from that anomaly of refraction known as "Astigmatism." He was not aware, he said, that he saw very differently from other people—although he knew his sight was somewhat defective. The walls of the buildings appeared to him often as if about to topple over, and he was sometimes even anxious about his safety from this cause. It was only after he was fitted with the proper glasses, by an oculist, that he discovered how badly he did see. To deprive him of those glasses now would be to deprive him of many, if not the majority, of the pleasures of life.

Persons who have suffered from myopia all their life, and have never had it corrected, after they have been fitted with the proper glasses, often remark that they had no conception of the beauty and loveliness of the world—for beyond a few inches, or, most, at a few feet, all was veiled in a mist.

The hyperepo, or farsighted person, who can read or apply himself to close work for a short time only, without fatigue, and who thinks his vision is in danger of being entirely lost, has suitable glasses given him for near work, and all his pain and fatigue vanishes as if by magic.

Again, these affections are often mistaken for amblyopia, commencing amaurosis, or even cataract. A patient applies to his physician complaining of an inability to see as well as he formerly did, especially near objects, and close application of the eyes gives him pain. He is not old enough for age to affect his vision, and if the physician is not expert in the use of the ophthalmoscope, he ascribes the trouble to the nerve, or retina, and informs his patient that he is, in all probability, suffering from a commencing amaurosis; that the disease will progress, and there is danger that he will ultimately lose his vision altogether. This, of course, has a bad influence on the *morale* of the patient; for the fact that he will probably go blind, is a heavy load for a man to carry in addition to the other burdens of life. Imagine the relief to this man when he finds that by the proper adaptation of glasses his vision can be made almost perfect, and that his optic nerve and retina are not diseased at all!

Within the last few years we have learned, also, that squint depends, in the majority of cases, upon some form of anomalous refraction; and if the child whose eyes are beginning to deviate has his error of refraction corrected the squint will be prevented. What estimate can be put upon such knowledge as this, especially in the case of young girls, to whom a deformity like this is such a sore affliction?

In regard to the completeness of relief in these cases, in no other department of medicine can we promise so much, where they are not of too high a degree, and uncomplicated with other incurable affections. Here we have reduced therapeutics to a mathematical certainty. The laws of optics are applied to diagnosis and treatment, and every thing is accomplished by the rules and principles of mathematics. In this particular, ophtholmology is far in advance of all the other branches of the healing art.

The affections that are generally reckoned among this class of eye diseases are *Myopia*, or shortsightedness; *Hypermetropia*, or farsightedness; *Presbyopia*, or old eyes; and *Astigmatism*, or an unequal refraction in different meridians of the cornea, and sometimes of the lens.

All of these, except Presbyopia, have this in common—that they depend upon an abnormal conformation of the eye-ball in some of its parts, and are, as I have before remarked, for the most part, congenital.

Hypermetropia and Presbyopia were, for a long time, and indeed, until quite recently, considered identical, and the terms were used, as can be seen by referring to some not yet obsolete books, synonymously. There is, however, a very essential and important difference between them; for they have been found to depend upon two entirely distinct morbid conditions.

But before we can clearly and correctly understand these diseased conditions, we must first have an idea of what a *normal*—or, as it is generally called, an *emmetropic* eye is.

Such an eye, in brief, is one in which, during rest of accommodation, parallel rays of light are brought to a focus upon the retina.

Even at the risk of repeating what, perhaps, many readers are already familiar with, I will explain what is meant by the term "accommodation" used in the preceding sentence, as it will materially assist us in understanding what follows.

It is well known that we possess the power to adapt our eyes for objects at different distances, the object only a few inches from us, and the landscape in the distance, both being distinctly seen, if our eyes are normal, *but not both at once.* If the eye is adapted for one, it is not adapted for the other; if viewing the landscape, or an object beyond twenty feet distance from us, it is adapted for *parallel* rays—(for all rays beyond twenty feet are parallel, even to an infinite distance); but, if looking at an object within the distance of twenty feet, it is accommodated for *divergent* rays, and the *more* divergent the *nearer the object.* Hence, we conclude that the eye must be possessed of some apparatus by which it can change the refraction of the rays of different degrees of divergence, in order that they may be brought to a focus upon the retina, and make a clear image. The eye *does* possess such an apparatus, and it is called the "accommodative apparatus."

I will not enter into the history of the different theories and speculations that have, from time to time risen, been adopted and thrown aside, each in turn, in explanation of this truly wonderful power resident in the eye; but will simply state that the ablest Ophthalmologists and Scientists of the present day consider that it is due to a change in the shape of the crystalline lens caused by the action of the ciliary muscle.

In viewing objects at a distance, the rays of light being parallel, the accommodation is not called into requisition; for, in the normal eye, as we have seen, its unaided refractive power is sufficient to bring parallel rays to a focus upon the retina. In looking at near objects, however, where the rays of light are divergent, if the direction of the rays were not altered, they would come to a focus *behind* the retina, and the image would be indistinct, but if the crystalline lens could be made *more convex* it would increase the refraction of the rays and bring them to a focus *upon* the retina and make a clear and distinct image. The nearer the object the more divergent will be the rays, and consequently the more convex must be the lens to produce a distinct image.

Accommodation for near objects, then, consists in an increased convexity of the lens, and the larger the amount of accommodation, the greater the convexity of the lens; and, as it is produced by muscular action, the greater must be the strain upon that muscle.

The distance from the point where there is no action of the accommodation to the nearest point of distinct vision, is called the "range of accommodation."

From this, we can readily see that any causes which would interfere with the action of the cilinary muscle, as paralysis, or weakening or stiffening from old age, would shorten this range of accommodation and remove the nearest point of distinct vision farther from the eye. Such, in fact, we find to be the case, and this condition being generally an accompaniment of old age is thence called *Presbyopia* (from *Presbus*, old; and *oph*, eye).

As in Hypermetropia, also, the eye cannot see near objects distinctly. The two terms, "Farsightedness" and "Presbyopia" were, as I have said, used synonymously. Farsightedness being, however, often met with in young persons, it would hardly be supposed that they could be identical. The researches of Denders and others, have indeed proven this to be the case, and shown that whereas Presbyopia depends upon an interference with the accommodation (generally by age), shortening its range and removing the near point farther from the eye, the far point remaining unchanged. Hypermetropia depends upon a shortening of the antero-posterior axis of the eye-ball—the range of accommodation not being materially affected, but both the far and near point being removed farther from the eye than is normal. In other words, while Presbyopia is an anomaly of *accommodation*, Hypermetropia is an anomaly of *refraction.* I hope to make this distinction more apparent when treating of the two affections separately.

In these articles it would be impossible to give the exact authority upon each particular point, but I will here state, in general terms, that we are indebted, for the greater part of our recent knowledge on the subject, to Prof. Denders, of Ulricht, to whose work ("Anomalies of Refraction and Accommodation," translated by the new Sydenham Society), I would refer those who desire a full and complete treatise on this interesting branch of Ophthalmology.

Prof. Helmholtz, now of Berlin, has also been a great and successful worker in this field, in addition to his labors in other branches of Physiological Science. Prof. Knapp, of New York, late of Heidelberg, has contributed considerably to our knowledge of these affections.

Without making any pretense to being exhaustive, we trust to give the busy, general practitioner some of the more recent views concerning this important class of eye-affections, which he may appropriate, and apply with benefit, in his practice.

